# Gene-level connections between anxiety disorders, ADHD, and head and neck cancer: insights from a computational biology approach

**DOI:** 10.3389/fpsyt.2025.1552815

**Published:** 2025-03-20

**Authors:** Meng Lian, Haiyang Li, Zhiyang Zhang, Jugao Fang, Xiaoqin Liu

**Affiliations:** ^1^ Department of Otorhinolaryngology Head and Neck Surgery, Beijing Tongren Hospital, Capital Medical University, Beijing, China; ^2^ Key Laboratory of Otorhinolaryngology Head and Neck Surgery, Capital Medical University, Ministry of Education, Beijing, China; ^3^ The National Clinical Research Center for Mental Disorders & Beijing Key Laboratory of Mental Disorders & Beijing Institute for Brain Disorders Center of Schizophrenia, Beijing Anding Hospital, Capital Medical University, Beijing, China; ^4^ Department of Otolaryngology, The Inner Mongolia Autonomous Region People’s Hospital, Hohhot, Inner Mongolia, China

**Keywords:** gene-level connections, anxiety disorders, ADHD, head and neck cancer, therapeutic exploration

## Abstract

**Background:**

Anxiety disorders (AD), ADHD, and head and neck cancer (HNC) are complex conditions with potential genetic interconnections that remain to be fully elucidated. The purpose of this study is to investigate gene-level connections among ADHD, AD, and HNC.

**Method:**

A comprehensive literature mining approach identified potential gene-disease relationships from PubMed and bioinformatics databases, analyzing 19,924 genes. An AI-driven computational process constructed a gene-disease relationship table using the Adjusted Binomial Method Algorithm (ABMA) to evaluate association reliability. Overlapping genes were analyzed through protein-protein interaction (PPI) networks, functional annotations, and literature-based pathway analyses to elucidate shared and unique genetic mechanisms linking these diseases.

**Results:**

The analysis identified 141 significant genes associated with AD, 153 with ADHD, and 1,065 with HNC (q-value < 0.05). These genes demonstrated significant overlap (odds ratio ≥ 1.8; p ≤ 2.58E-2) and high interconnectivity (PPI network density ≥ 0.39, clustering coefficient ≥ 0.76, and diameter ≤ 3). Centrality analysis revealed core genes such as IL-6, MYC, NLRP3, and CXCR4 as critical mediators. Functional enrichment analysis identified key pathways, including serotonergic synapse, inflammatory response, and Toll-like receptor signaling, highlighting the involvement of neuronal and immune mechanisms. Functional pathway analysis demonstrated reciprocal genetic influences among AD, ADHD, and HNC, emphasizing shared and distinct gene-level connections that may underlie their co-occurrence and mutual risk factors.

**Conclusion:**

This study reveals a complex and interconnected genetic network among AD, ADHD, and HNC, highlighting shared pathways, unique mechanisms, and critical genes, providing valuable insights into the genetic underpinnings of these conditions and potential avenues for therapeutic exploration.

## Introduction

Anxiety disorders (AD) are a group of mental health conditions characterized by excessive fear, worry, and related behavioral disturbances. Epidemiologically, ADs are highly prevalent, affecting approximately 19.1% of adults in the United States in a given year, with a lifetime prevalence of around 29% (1). These disorders can arise from a combination of genetic, environmental, and psychological factors, making understanding their epidemiology crucial for effective prevention and treatment strategies (2).

Attention-Deficit/Hyperactivity Disorder (ADHD) is a neurodevelopmental disorder characterized by persistent patterns of inattention, hyperactivity, and impulsivity that can interfere with functioning or development. ADHD affects approximately 6-7% of children worldwide (3), with varying prevalence rates across different regions and populations.

Head and Neck Cancer (HNC) encompasses a diverse group of malignancies that arise in the oral cavity, pharynx, larynx, and other related structures. Epidemiologically, HNC accounts for approximately 4% of all cancers in the United States, with an estimated 54,540 new cases and 10,780 deaths projected for 2023 (4).

To clarify terminology, AD in this study refer to the broad spectrum of anxiety-related conditions as defined by the Diagnostic and Statistical Manual of Mental Disorders (DSM-5), including but not limited to generalized anxiety disorder (GAD), panic disorder, separation anxiety disorder, and social anxiety disorder. While these subtypes differ in their specific symptomatology, they share core features of excessive fear, worry, and behavioral disturbances. We use the term AD to encompass this spectrum, acknowledging that this inclusive approach may obscure subtype-specific nuances but allows for a broader exploration of shared genetic mechanisms. Importantly, AD in this context refers specifically to Anxiety Disorders and not Alzheimer’s disease, which is another psychiatric condition with distinct pathophysiology and clinical presentation.

Similarly, HNC encompasses a diverse group of malignancies arising in the oral cavity, pharynx, larynx, sinuses, and salivary glands. This includes squamous cell carcinomas, adenocarcinomas, and other histological subtypes. By using the term HNC, we aim to capture the shared genetic and clinical features across these malignancies, recognizing that this broad categorization may overlook subtype-specific differences. In this study, gene-disease associations were analyzed at the level of full clinical diagnoses rather than individual symptoms to ensure consistency across ADHD, AD, and HNC. While ADHD is generally treated as a single diagnostic entity, AD and HNC encompass multiple subtypes with distinct but overlapping genetic underpinnings. While this approach facilitates a comprehensive analysis of shared genetic pathways, it also necessitates caution in interpreting findings, as the heterogeneity within HNC subtypes may influence results.

Associations have been suggested between ADHD and AD, with ADHD often co-occurring with some form of anxiety (5). Genome-wide association studies have demonstrated significant genetic correlations between ADHD and AD (rg = 0.34), with both conditions sharing genetic risks linked to neuroticism (rg = 0.81) and major depressive disorder, suggesting a common polygenic architecture that may explain their frequent comorbidity (6, 7). Mendelian randomization analyses further reveal that socioeconomic factors, such as higher educational attainment and income, serve as protective influences for both ADHD and anxiety disorders, underscoring the role of gene–environment interactions in their co-occurrence (8). Together, these findings highlight the importance of exploring the shared and unique genetic factors underlying these conditions.

Extending this framework to HNC involves investigating shared biological pathways such as neuroinflammation and immune dysregulation. In HNC patients, higher pretreatment anxiety levels are significantly associated with poorer 2-year overall survival, with tumor response mediating this relationship, suggesting that AD may negatively impact cancer outcomes (9). Conversely, HNC patients, particularly those who have undergone radiotherapy, may develop anxiety and depressive disorders (10). Moreover, shared genes have been identified as playing roles in all three disorders, including CYP2D6 (11–13). Emerging hypotheses propose that systemic inflammation—implicated in ADHD and anxiety through genetic variants in pathways like IL6 and TNF-α—may also contribute to oncogenic processes in HNC (14, 15). For instance, chronic inflammation and oxidative stress, common in neuropsychiatric conditions, are established drivers of carcinogenesis. Although direct genetic links between HNC and psychiatric disorders remain underexplored, the overlap in inflammatory pathways provides a plausible mechanistic bridge. Additionally, while ADHD-associated behaviors (e.g., tobacco use) may elevate HNC risk, genetic predispositions to immune dysregulation could further compound susceptibility (16). By elucidating these shared mechanisms, research may uncover transdiagnostic therapeutic targets and inform preventive strategies across neurodevelopmental, psychiatric, and oncological conditions.

This study aims to address the gap in knowledge by exploring gene-level connections among these conditions using a computational biology approach. We hypothesize that there are significant overlapping genetic pathways and core genes that contribute to the co-occurrence and mutual risk factors of AD, ADHD, and head and neck cancer. The findings of this study could provide valuable insights into the genetic underpinnings of these conditions, potentially informing future research and therapeutic strategies.

## Method

### Study workflow

**Figure 1:**
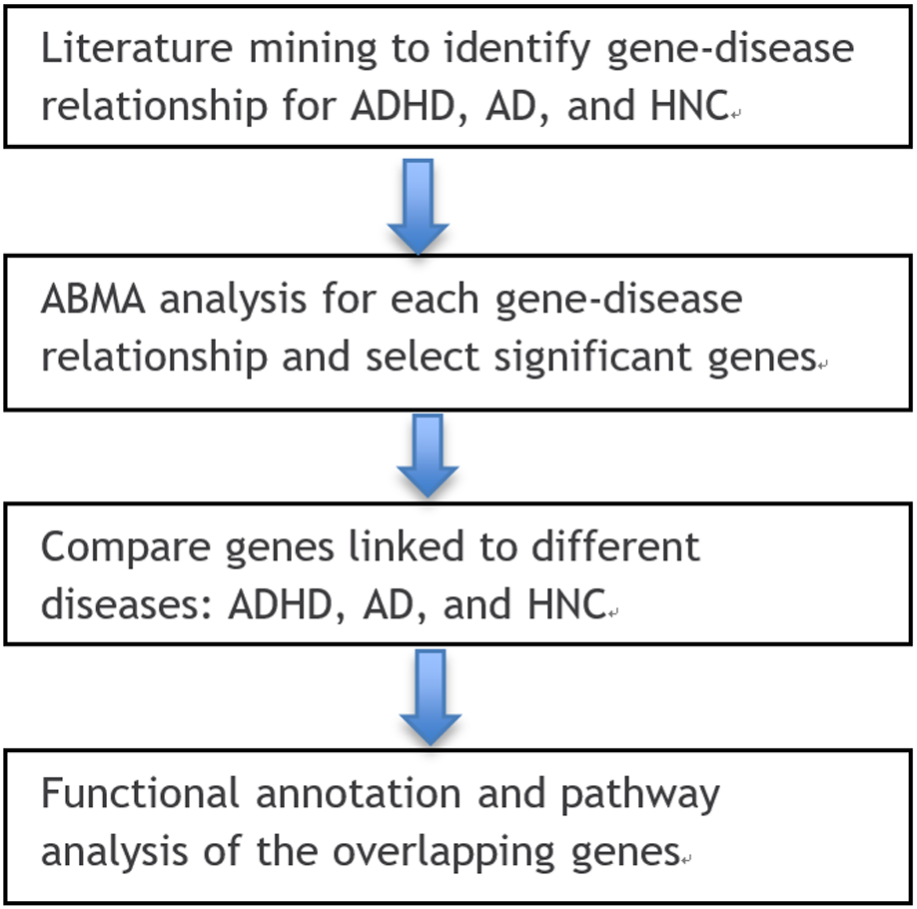
Workflow diagram of this study. Generated using https://bioinfogp.cnb.csic.es/tools/venny/.

This study followed a structured multi-step workflow to explore the genetic relationships among ADHD, AD, and HNC. First, relevant gene-disease associations were retrieved from multiple bioinformatics databases and literature sources, including PubMed and the AIC Bioinformatics Database (ABD). Next, an AI-driven data processing pipeline was applied to filter and refine gene-disease associations, ensuring high-quality data for subsequent analysis. Overlapping and unique gene sets across the three diseases were identified, and statistical enrichment analyses were conducted to assess their biological significance. Finally, functional and pathway analyses were performed to investigate potential mechanisms linking ADHD, AD, and HNC. Details of each step, including data sources, computational approaches, and statistical methods, are provided in the subsequent sections. The workflow of the current study is depicted in **Figure 1**. To note, as described in the Introduction, AD refers to anxiety disorders as defined by DSM-5, and HNC encompasses multiple malignancies arising in the head and neck region. This study considers ADHD as a single diagnostic entity, while AD and HNC include multiple subtypes with shared genetic features.

### Disease gene identification using literature-based mining

To systematically identify gene-disease associations for ADHD, AD, and HNC, we conducted a comprehensive literature mining approach utilizing multiple bioinformatics resources. The analysis encompassed whole-genome genes (19,924 genes) and aimed to extract relevant disease-gene relationships from curated scientific literature.

First, we employed the Entrez API (http://www.ncbi.nlm.nih.gov/Entrez/) to programmatically query PubMed (https://pubmed.ncbi.nlm.nih.gov) for relevant publications. This automated process retrieved references containing associations between the three diseases and specific genes. The extracted information included metadata such as PMID, DOI, publication title, abstract, author details, and publication date, ensuring a structured dataset for downstream analysis.

Additionally, we leveraged the AIC Bioinformatics Toolbox (ABT) to extract disease-gene relationships from the AIC Bioinformatics Database (ABD) (https://www.gousinfo.com/en/userguide.html). This database integrates information from various genomic and biomedical data sources, facilitating a more comprehensive collection of gene-disease associations. The data were formatted to ensure uniformity and compatibility with PubMed-extracted references.

The retrieved reference data were compiled and formatted into an Excel worksheet, where each entry was cross-referenced for relevance and quality. Further post-processing steps, including data filtering, duplicate removal, and relevance scoring, were performed to refine the dataset before proceeding with statistical and functional analyses. To ensure consistency, gene-disease associations were analyzed at the level of full diagnoses rather than individual symptoms. While AD comprises multiple subtypes with distinct diagnostic criteria, only studies explicitly linking genetic markers to diagnosed AD subtypes were included in the analysis. Similarly, HNC subtypes were collectively analyzed, focusing on shared genetic components rather than subtype-specific variations.

### AI-based relationship table construction

For the references identified, an AI-based quality control process was used to extract relationship details and remove non-qualified references. The relationship between each gene-disease pair was then constructed, with polarity and direction assigned using an AI-driven computational approach. To assess the reliability of these relationships, the Adjusted Binomial Method Algorithm (ABMA) was applied using the scipy.stats package in Python, which offers functions for hypothesis testing. The core idea of ABMA is to refine the estimation of association strength by adjusting for different types of observations—positive, negative, and inconclusive findings. This ensures that the influence of each type of observation is properly accounted for in the final association score. ABMA assumes that the proportions of positive, negative, and inconclusive findings are representative of the underlying relationships between the gene and the disease. The method also adjusts for uncovered samples based on an estimated factor (α), which is assumed to be a reasonable estimate of missing data. While this approach helps refine the association estimation, any inaccuracies in the assumptions (e.g., the coverage of uncovered samples or the distribution of findings) could influence the final results. A False Discovery Rate (FDR) correction was applied to control for false positives, and relationships with a q-value ≤ 0.05 were considered significant.

### Adjusted binomial method algorithm

To assess the association between two entities, such as a gene and a disease, we implemented the Adjusted Binomial Method algorithm, which applies an adjusted binomial test to evaluate statistical significance. This method accounts for multiple types of observations—positive, negative, and inconclusive findings—to provide a refined estimation of association strength.

The algorithm was executed using the scipy.stats package in Python (https://docs.scipy.org/doc/scipy/reference/stats.html), which offers statistical functions for hypothesis testing. The key principle behind this method is to determine whether the observed proportion of a dominant outcome (e.g., positive associations between a gene and a disease) significantly deviates from an expected probability threshold (p₀).

### Total observations calculation

The total sample size N is calculated using the following formula:


$$N\;=\;n_{p}\;+\;n_{n}\;+\;n_{0}\;+\;n_{x}$$

where, N represents the total effective sample size, $n_{p}$ , $n_{n}$ , and $n_{0}$ represent the sample size of positive, negative, or unknown relationship, and $n_{x}$ represent uncovered samples.

where $n_{p}$ , $n_{n}$ , and $n_{0}$ represent the number of studies reporting a positive, negative, or unknown association, respectively, while $n_{x}$ represents the number of uncovered samples. To account for potential publications not identified through the initial search, we use an uncovered sample fraction factor α , which represents the ratio of uncovered to covered samples:


$$n_{x}=\alpha \;*\;\left(n_{p}\;+\;n_{n}\;+\;n_{0}\right)$$

For this study, the fraction factor α is set to 1. This choice is based on the assumption that PubMed and the ABD database together provide comprehensive coverage of bioinformatics and biology studies, capturing around 50% of publications in the field. Therefore, assuming uncovered samples to be at most equal to identified samples is a reasonable estimate.

### H0 testing using adjusted binomial test

Null Hypothesis (H0): The true proportion of major results is equal to p0.

Alternative Hypothesis (H1): The true proportion of positive results >p0.

Decision rule: If the calculated p-value is less than or equal to the significance level (<0.05), the null hypothesis is rejected, indicating a statistically significant association (**Table 1**). In the following description, we use positive association as the dominant finding as example.

**Table 1:** Venn diagram statistics for overlapping genes among three diseases.

Gene Category	Source Disease	Target Disease	#genes Source	#genes Target	Overlap	Odds ratio	p-value
Significant Genes (q-value ≤ 0.05)	Anxiety disorders	ADHD	141	153	32	36.80	1.24e-34
	Anxiety disorders	HNC	141	1065	22	3.12	1.36E-05
	ADHD	HNC	153	1065	15	1.8	2.58E-02
All genes	Anxiety disorders	ADHD	2301	1199	723	7.15	1.31E-252
	Anxiety disorders	HNC	2301	6629	1521	3.91	1.34E-201
	ADHD	HNC	1199	6629	766	3.54	1.64e-97

The tail probability was calculated using the function below:


$$\;p-value=\;P\left(X\;\geq \;n_{p}\right)\;=\;binom.sf\left(n_{p}\;-\;1,\;N,\;p0\right)$$

where binom.sf is the survival function for a binomial distribution with N trials and success probability p0.

To determine the total number N , the success probability p0 , we consider the following two cases:

1) Case 1: We hypothesize that the uncovered $n_{x}$ samples have the same distribution as the identified samples. Under this hypothesis, the adjusted total number of observations is:


$$N\;=\;2\;*\;(n_{p}\;+\;n_{n}\;+\;n_{0})$$

with the number of dominant findings is doubled:


$$n_{p}=2\;*\;n_{p}$$

In this case, the observed proportion of dominant associations out of the total adjusted observations is:


$$\;n_{obser}\;=\;\frac{n_{p}}{N}\;\in \left(0.33,1\right]$$

Based on this range, we set p0 =  0.34, which represents the lower bound at which $n_{p}$ dominates the observations.

2) Case 2: We hypothesize that the uncovered $n_{x}$ ​ samples are different from the identified samples. In the extreme scenario, all $n_{x}$ samples are null associations. Here, the adjusted total number of observations is:


$$N\;=\;2\;*\;(n_{p}\;+\;n_{n}\;+\;n_{0})$$

and the number of dominant findings $n_{p}$ does not change. Therefore, the observed proportion of dominant associations out of the total adjusted observations is:


$$\;n_{obser}\;=\;\frac{n_{p}}{N}\;\in \left(0.17,1\right]$$

Therefore, in this case, we set p0 =  0.17, which represents the lower bound at which $n_{p}$ dominates the observations.

### Gene comparison across diseases

The gene lists associated with each of the three diseases—ADHD, AD, and HNC—were compared to identify unique and overlapping genes. Fisher’s exact test was used to assess the significance of the overlap, and a Venn diagram was employed for visualization. While we compared both all disease-related genes and those that were statistically significant, our subsequent analysis will focus primarily on the genes showing statistical significance.

### Functional analysis of overlapping genes

We employed the Functional Annotation Tool of Database for Annotation, Visualization, and Integrated Discovery (DAVID) (https://david.ncifcrf.gov) to systematically analyze the overlapping gene set. The functional annotation covered Gene Ontology (GO) Analysis: Biological Process (BP) (GOTERM_BP_DIRECT), investigating the biological roles of genes (e.g., immune response, neurodevelopment, apoptosis); Cellular Component (CC) (GOTERM_CC_DIRECT), identifying subcellular localization (e.g., nucleus, cytoplasm, synapse); and Molecular Function (MF) (GOTERM_MF_DIRECT), examining gene product functions (e.g., kinase activity, DNA binding). Additionally, Pathway Enrichment Analysis was conducted: BBID Pathway, exploring regulatory interactions; BIOCARTA Pathway, providing manually curated molecular interactions; and KEGG Pathway, identifying involvement in well-defined biological pathways (e.g., cancer, metabolism, neurodevelopment).

Additionally, a protein-protein interaction (PPI) analysis was performed to explore functional connections between these genes, with relationships between proteins established based on prior literature.

Finally, a functional pathway analysis was conducted to construct potential associations among ADHD, AD, and HNC at the gene level. Gene interactions were identified based on known biological pathways and established functional connections in curated databases. The analysis does not infer direct causation but highlights potential regulatory relationships that could mediate interactions between these conditions. While gene expression and functional annotation suggest possible influence, experimental validation is necessary to establish causal mechanisms.

## Results

### AI-based disease-gene identification results

**Figure 2:**
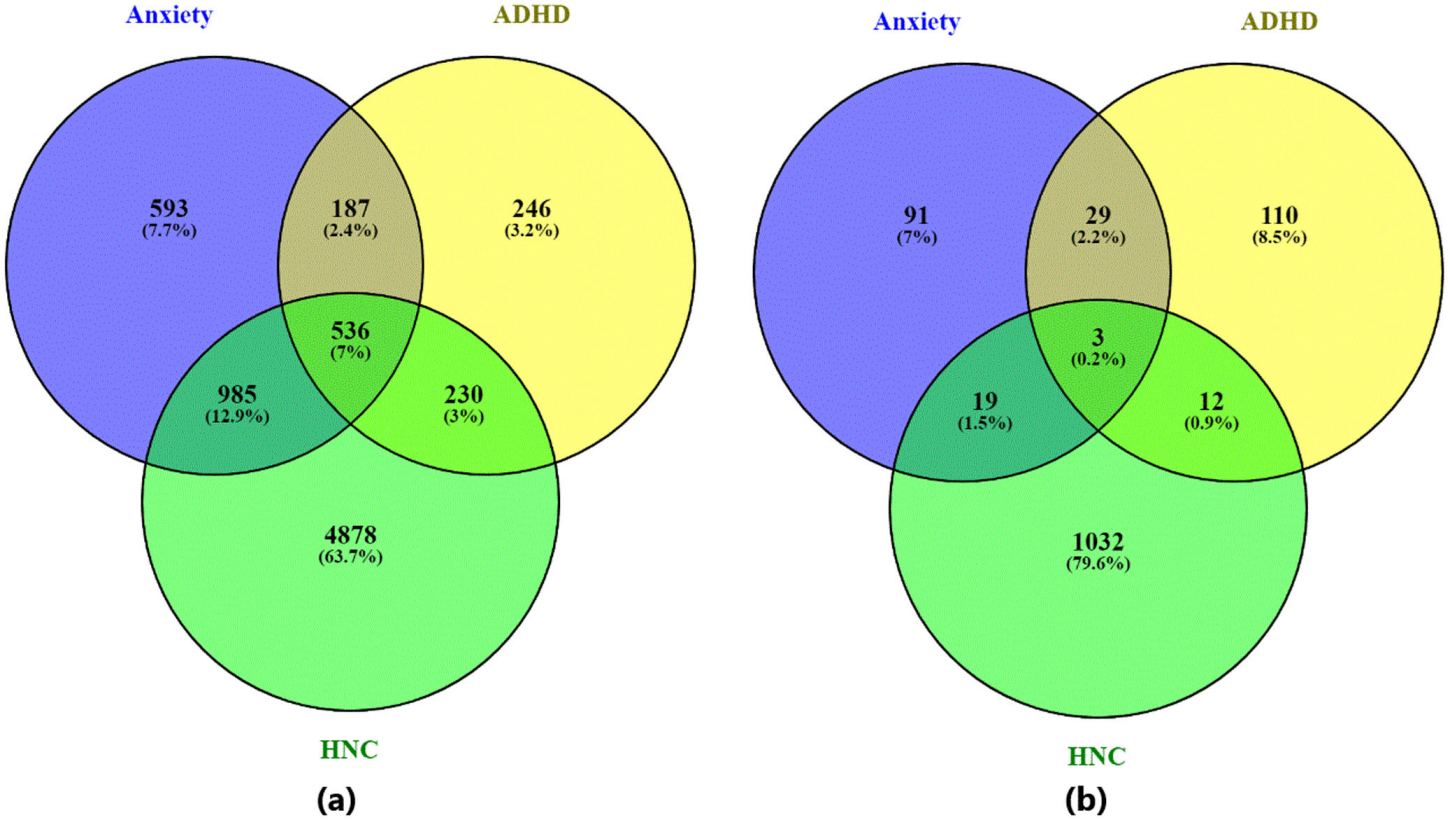
Venn diagram illustrating the overlap between genes associated with the three diseases—ADHD, anxiety disorder, and head and neck cancer. **(a)** Venn diagram based on all identified disease-related genes; **(b)** Venn diagram based on statistically significant disease-related genes (q-value ≤ 0.05). Generated using visualize_pathwayInExcel() function within NetworkAnalysis.py.

Out of 19,924 genes, our AI-based computational approach identified 2,301 genes associated with Anxiety (supported by 4,884 references), 1,199 genes associated with ADHD (supported by 3,283 references), and 6,629 genes associated with HNC (supported by 17,477 references) (see **Figure 2a**). When applying a significance threshold (q-value ≤ 0.05), 141 genes were identified for Anxiety (784 references), 153 for ADHD (1,005 references), and 1,065 for HNC (4,458 references) (see **Figure 2b**).

The overlap analysis between Anxiety and ADHD indicates a statistically significant association. For the set of significant genes (q-value ≤0.05), 32 overlapping genes were identified, yielding an odds ratio of 36.80 and a p-value of 1.24e-34. When considering all identified genes, 723 genes overlapped, with an odds ratio of 7.15 and a p-value of 1.31E-252. These results indicate a consistent association between AD and ADHD.

In comparison, the overlap between Anxiety and HNC showed 22 significant overlapping genes with an odds ratio of 3.12 and a p-value of 1.36e-05 (**Table 1**). For all genes, the overlap increased to 1,521 genes, with an odds ratio of 3.91 and a p-value of 1.34e-201. This suggests a statistically significant, although less pronounced, genetic overlap between AD and HNC.

For the ADHD and HNC comparison, 15 overlapping significant genes were identified (odds ratio = 1.80, p-value = 2.58e-02) (**Table 1**). When all genes were considered, 766 overlapping genes were observed, with an odds ratio of 3.54 and a p-value of 1.64e-97. While these associations are statistically significant, they are relatively weaker compared to the overlaps involving Anxiety and ADHD.

### PPI analysis

#### ADHD and HNC

**Figure 3:**
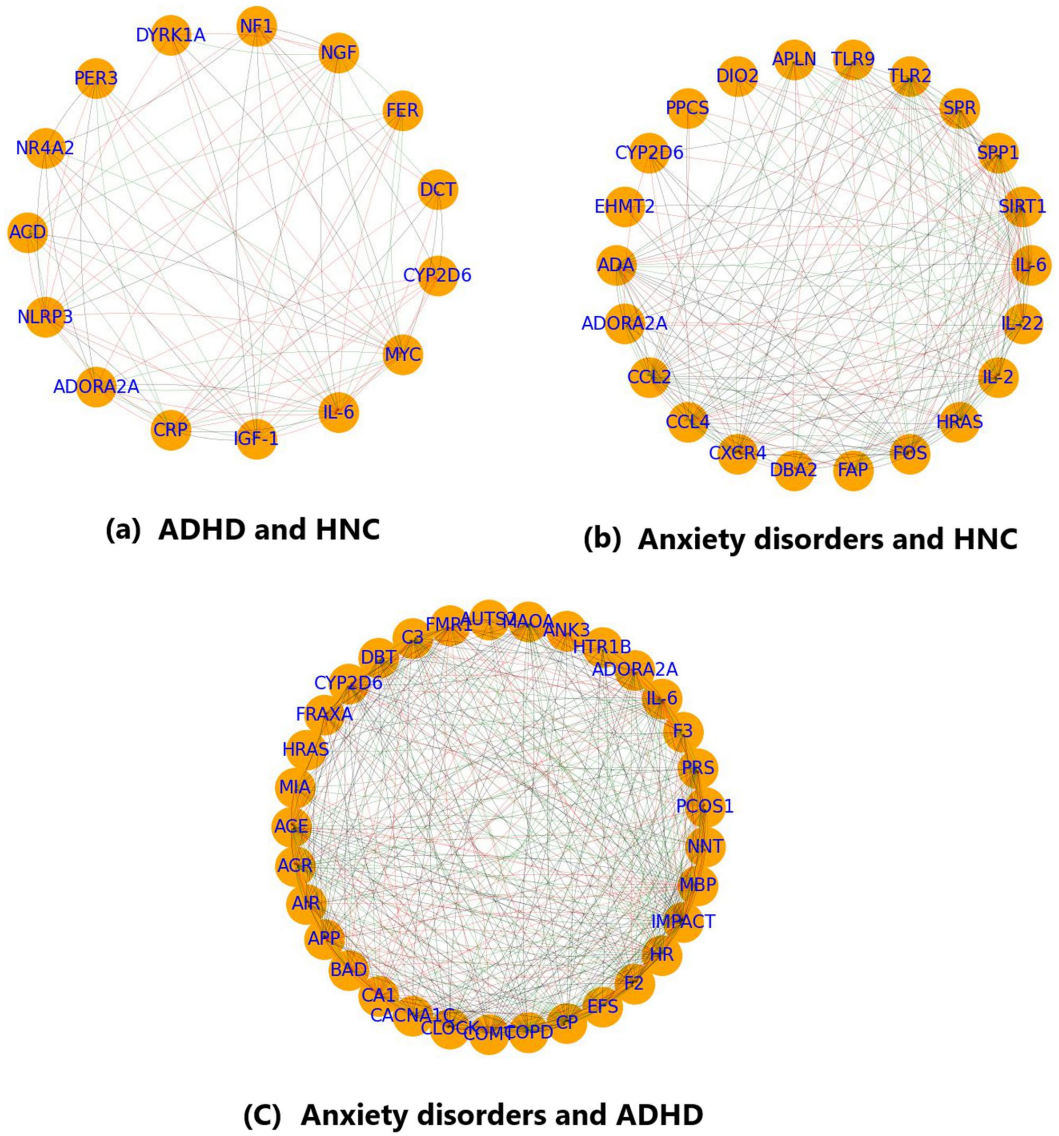
PPI analysis showing the interplay between the overlapping genes. **(a)** PPI network of the genes shared by ADHD and HNC; **(b)** PPI network of the genes shared by Anxiety disorder and HNC; **(c)** PPI network of the genes shared by Anxiety disorder and ADHD. Generated using excel file with data from Enrichment analysis (see Functional Annotation.xlsx).

The PPI analysis for the 15 overlapping genes between ADHD and HNC (including *ACD, ADORA2A, CRP, CYP2D6, DCT, DYRK1A, FER, IGF-1, IL-6, MYC, NF1, NGF, NLRP3, NR4A2*, and *PER3*) produced a network with 15 nodes and 87 edges (see **Figure 3a**). The network has a density of 0.41, an average path length of 1.67, an average clustering coefficient of 0.79, one connected component, and a diameter of 2. The Total Weight of the network is 304 (based on supporting references). Centrality analysis identified five genes (*IL-6, MYC, NLRP3, IGF-1*, and *CRP*) as network hubs, based on consistently high centrality measures.

#### Anxiety and HNC

The PPI network for overlapping genes associated with Anxiety and HNC consists of 22 nodes and 201 edges (see **Figure 3b**). This network exhibits a density of 0.44, an average path length of 1.63, a diameter of 2, and an average clustering coefficient of 0.85, indicating moderate connectivity with a single connected component. The Total Weight is 757. Key genes based on centrality metrics include SIRT1, IL-6, CXCR4, CCL2, FOS, and TLR2, with IL-6 showing the highest betweenness centrality (0.18).

#### ADHD and anxiety

For the overlapping genes between ADHD and Anxiety, the PPI network comprises 33 nodes and 411 edges (see **Figure 3c**). The network density is 0.39, the average path length is 1.69, and the average clustering coefficient is 0.76, with one connected component and a network diameter of 3. The total edge weight is 1,190. Centrality analysis highlighted IL-6, IMPACT, COPD, HR, and COMT as prominent nodes. In particular, IL-6 exhibited high in-degree (0.62), out-degree (0.78), and a betweenness centrality of 0.16, while COMT had a notable eigenvector centrality (0.23).

### Functional annotation analysis results

#### Anxiety and ADHD

**Figure 4:**
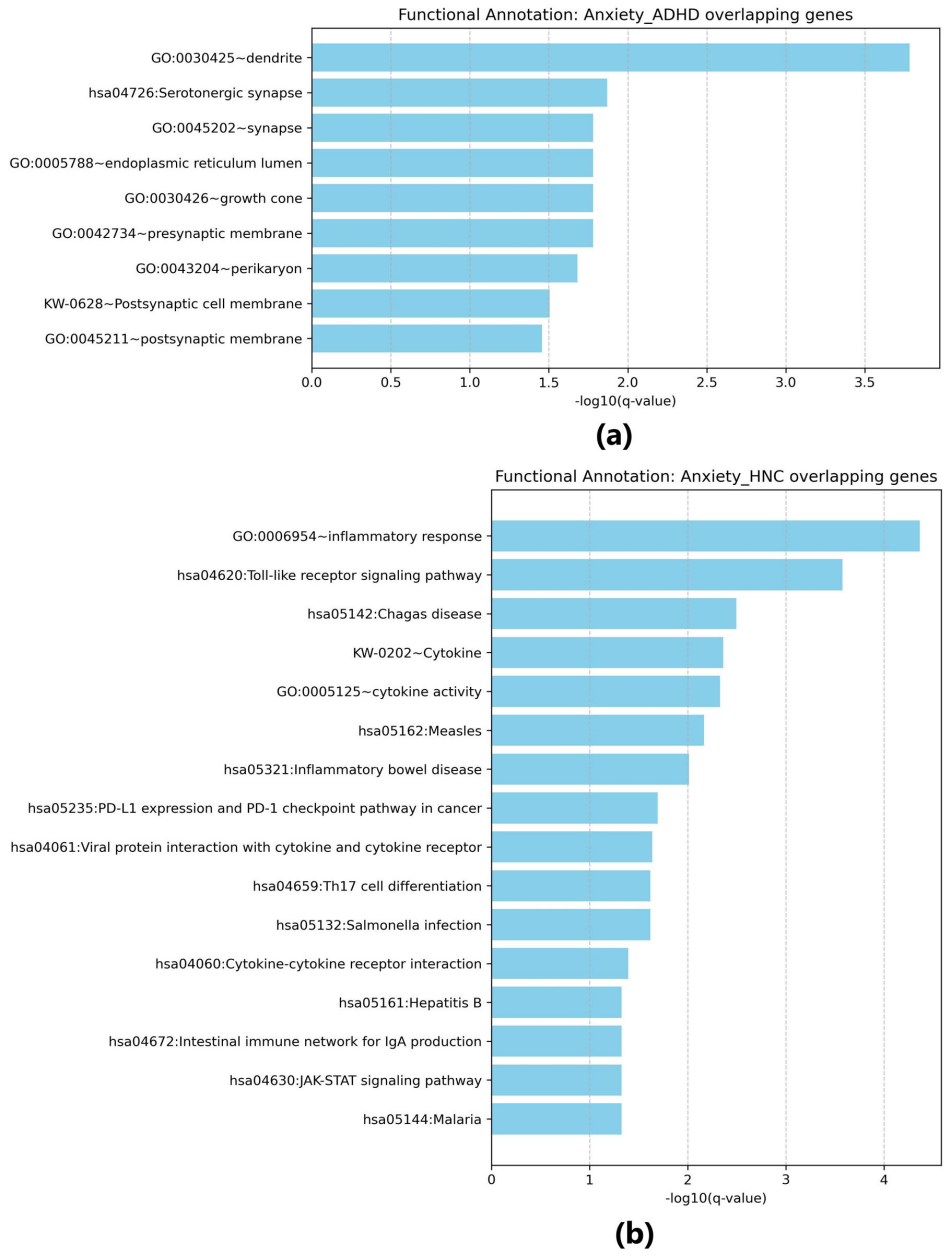
Functional enrichment analysis for overlapping genes. **(a)** Results for overlapping genes associated with both Anxiety disorders and ADHD; **(b)** Results for overlapping genes associated with both Anxiety disorders and head and neck cancers. Generated using Cytoscape.

The functional enrichment analysis for overlapping genes between Anxiety and ADHD (**Figure 4a**) identified several significant biological terms and pathways. For example, the term “dendrite” (GO:0030425) was enriched (p = 1.46e-06) with a fold enrichment of 12.97, involving genes such as APP, ADORA2A, and CACNA1C. The “Serotonergic synapse” pathway (hsa04726) was also enriched (p = 9.06e-05) with a fold enrichment of 19.22, including genes such as MAOA and HTR1B. Additional terms such as “synapse” (GO:0045202, p = 4.21e-04), “endoplasmic reticulum lumen” (GO:0005788, p = 6.46e-04), “growth cone” (GO:0030426, p = 7.20e-04), and “presynaptic membrane” (GO:0042734, p = 7.35e-04) were significantly enriched, emphasizing the involvement of neuronal structure and signaling.

#### Anxiety and HNC

For overlapping genes between Anxiety and HNC (**Figure 4b**), functional enrichment analysis identified terms such as “inflammatory response” (GO:0006954, p = 7.85e-08), involving genes like IL22, IL6, and CXCR4, and the “Toll-like receptor signaling pathway” (hsa04620, p = 1.96e-06) with contributions from IL6 and TLR9. Additional enriched pathways include “Chagas disease” (hsa05142, p = 4.69e-05) and “Cytokine-cytokine receptor interaction” (hsa04060, p = 0.00266). Other immune-related pathways, such as “Th17 cell differentiation” (hsa04659, p = 0.00127) and “Cytokine activity” (GO:0005125, p = 3.47e-05), were also noted, with fold enrichment values up to 28.63 (e.g., for “Inflammatory bowel disease”, hsa05321). These findings point to a significant association with immune and inflammatory responses in the overlapping genes.

### Pathway connecting AD, ADHD, and HNC

**Figure 5:**
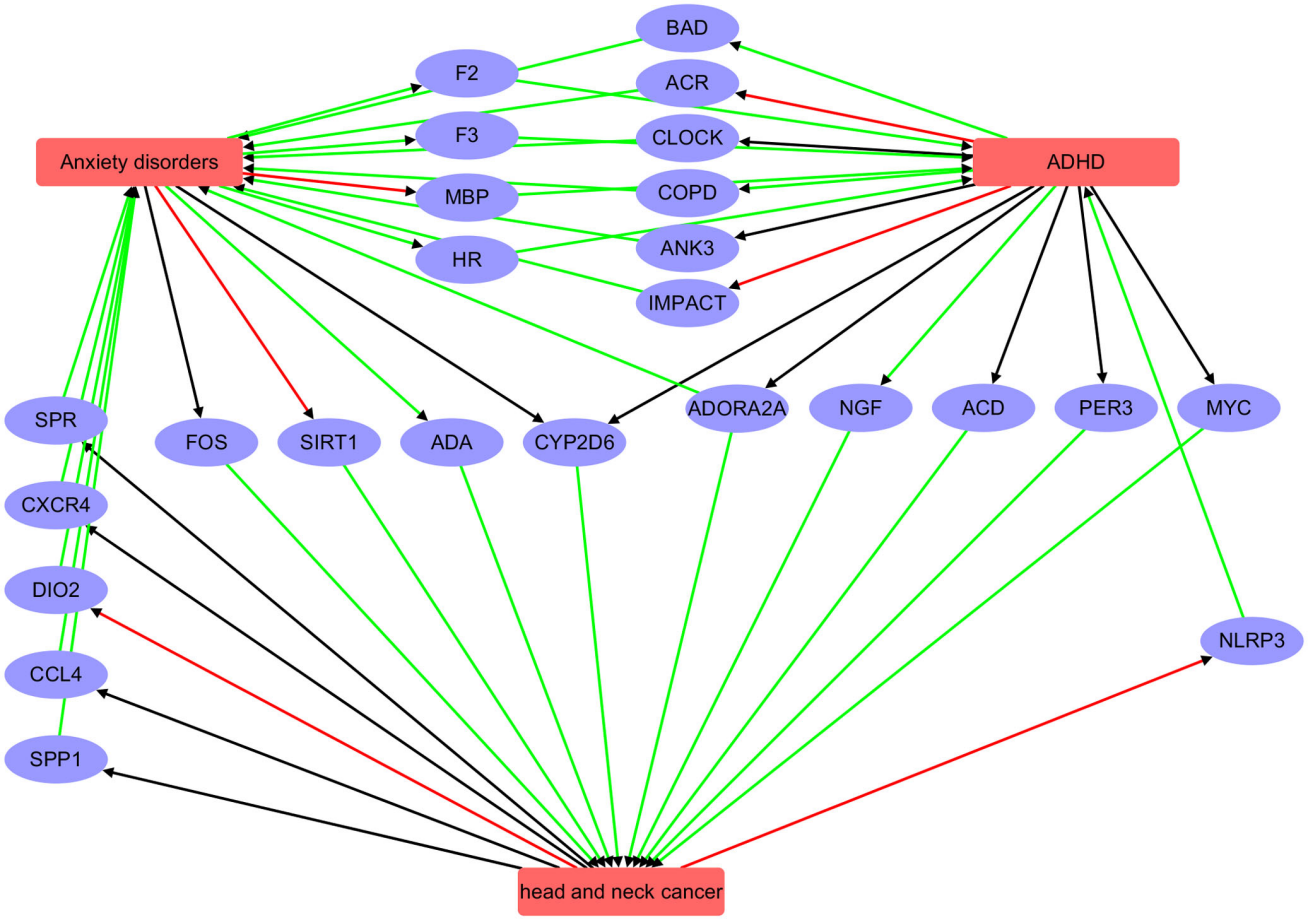
Pathway connecting three diseases: Anxiety disorder, ADHD, and head and neck cancer. Red edge indicates a negative association, green positive association, and black unknown association.

The functional pathway analysis (**Figure 5**) indicates that ADHD, AD, and head and neck cancer (HNC) may influence each other through the regulation of multiple genes.

AD and ADHD: AD may influence ADHD via intermediary genes such as F2, HR, F3, and MBP. Conversely, ADHD may affect AD through genes including CLOCK, ACR, BAD, ADORA2A, ANK3, COPD, and IMPACT.

AD and HNC: AD appear to affect HNC through genes such as CYP2D6, ADA, FOS, and SIRT1, while HNC may influence AD via genes like SPR, CCL4, CXCR4, DIO2, and SPP1.

ADHD and HNC: ADHD may impact HNC through genes such as CYP2D6, PER3, ACD, MYC, and NGF, although the reciprocal influence of HNC on ADHD was not evident.

Overall, these pathway analyses reveal a complex genetic network that may underlie the co-occurrence or shared risk factors among these three conditions. While these findings suggest potential regulatory pathways, they do not establish direct causation. Further experimental studies, such as gene expression validation and mechanistic investigations, are needed to confirm these inferred relationships.

## Discussion

The study employs an AI-based computational approach to identify genes associated with AD, ADHD, and head and neck cancer (HNC) from a pool of 210,741 genes. It reveals significant gene overlaps among these diseases, suggesting shared genetic factors. The analysis identifies 2,301 genes linked to anxiety, 1,199 to ADHD, and 6,629 to HNC, with statistically significant overlaps and high odds ratios, particularly between anxiety and ADHD. Protein-protein interaction (PPI) networks highlight core genes like IL-6 and SIRT1, indicating robust connectivity and potential shared pathways. Functional enrichment analysis underscores the involvement of synaptic, immune, and inflammatory processes. The study suggests complex genetic networks linking these conditions, with shared genes potentially contributing to co-occurrence or mutual risk factors.

Functional enrichment analysis underscores the involvement of synaptic, immune, and inflammatory processes. For instance, a study identified 181 out of 235 genes associated with ADHD that were enriched in 100 pathways, highlighting multiple associations with ADHD (17). Additionally, a genome-wide association study revealed shared genetic components between tinnitus and psychiatric disorders, such as bipolar disorder, suggesting common pathways (18). These findings suggest complex genetic networks linking these conditions, with shared genes potentially contributing to co-occurrence or mutual risk factors.

The current study identifies a significant overlap of 32 genes between AD and ADHD, with an odds ratio of 510.68, indicating a substantial genetic association. Previous studies have also suggested shared genetic factors, but the magnitude of overlap and specific genes involved may vary, with some research highlighting different sets of candidate genes or weaker associations. Conflicts may arise from variations in sample sizes, methodologies, and the specific populations studied, which can lead to differing conclusions about the extent and nature of the genetic links between these disorders. For instance, a study on Lebanese populations highlighted various factors associated with ADHD, such as maternal stress and familial history, analyzed using Fisher’s exact test, but did not focus on genetic overlap with AD (19). Another study found a significant association between ADHD and psychiatric disorders in patients with epilepsy, again using Fisher’s exact test, but did not explore genetic links with AD (20). In contrast, studies on AD have shown significant associations with other conditions, such as impulse control disorders in Parkinson’s disease, using Fisher’s exact test, but did not delve into genetic overlaps with ADHD (21).

The current study identifies 22 overlapping genes between AD and HNC, with a significant odds ratio of 37.14. This statistically significant genetic overlap suggests that, despite the distinct clinical presentations of psychiatric disorders and malignancies, shared biological pathways may underlie their co-occurrence. Prior research has shown that chronic psychological stress—a central feature of AD—can alter neuroendocrine function and modulate immune responses, thereby influencing cancer progression (22, 23). For example, stress-induced dysregulation of cortisol and catecholamine levels can activate inflammatory pathways that promote tumorigenesis. At the same time, other studies have reported distinct genetic mechanisms involved in the pathophysiology of AD and HNC, underscoring the complexity of these associations. These findings highlight the need for further research to elucidate the specific molecular and cellular mechanisms linking AD with HNC.

This finding also aligns with previous research that has linked anxiety not only to other neuropsychiatric conditions (24) and cognitive impairments (25) but also to adverse clinical features in cancer. In the context of HNC, several studies have reported that patients experiencing anxiety are more likely to report chronic pain after radiotherapy (26) and that higher anxiety levels are associated with socioeconomic deprivation, which may further influence treatment adherence and overall outcomes (27). The use of AI-based computational approaches in our study offers a novel perspective on these genetic overlaps, suggesting that the shared genetic factors may partially underlie these clinical associations. However, while our findings point to a statistically significant genetic link, the specific pathways—such as those mediating inflammatory responses or neuroendocrine dysregulation—remain to be fully delineated. Therefore, further investigation is warranted to clarify these mechanisms and to explore their implications for the treatment and management of both AD and HNC.

The identification of 15 overlapping genes between ADHD and HNC, with an odds ratio of 21.69, suggests a previously unexplored genetic link between these conditions. While prior research has examined their individual genetic and environmental factors, potential genetic intersections have received little attention. For example, some studies have primarily focused on associations with factors such as maternal anemia, stress, and familial history in ADHD (19), and genetic predispositions in HNC, such as the protective role of certain VEGF alleles (28). Our findings highlight the need for further investigation into shared biological pathways, which may provide new insights into the underlying mechanisms connecting neurodevelopmental and oncogenic processes.

AD and ADHD share several neurobiological pathways that contribute to their comorbidity, particularly those involving the serotonergic system, synaptic plasticity, and neuronal connectivity. Dysregulation of serotonergic signaling, crucial for mood regulation and cognitive functions, has been implicated in both disorders, with ADHD linked to alterations in circuits involving the Pet-1 transcription factor and Cadherin-13, affecting serotonin neuron migration and synaptic balance (29, 30). Similarly, AD benefit from treatments targeting serotonergic synapses, such as selective serotonin reuptake inhibitors, emphasizing the shared importance of this pathway (31). Beyond serotonin, both conditions exhibit disruptions in synaptic and dendritic processes. ADHD is associated with synaptic dysfunction and altered neurotransmitter release (32), while AD involve changes in synaptic plasticity and dendritic spine morphology, affecting emotional regulation and cognition (33). Additionally, disruptions in protein processing within the endoplasmic reticulum lumen may impair neurotransmitter release, further linking the two disorders through impaired synaptic transmission. These shared pathways suggest that neurodevelopmental and emotional dysregulations in ADHD may predispose individuals to AD, with a common neurobiological substrate underlying their comorbidity. Understanding these mechanisms could guide the development of targeted therapies addressing the overlapping neurobiological features of AD and ADHD.

AD may influence the development and progression of HNC through several biological mechanisms, including immune dysregulation and the promotion of a pro‐inflammatory state. Chronic anxiety is associated with altered cytokine profiles—such as elevated levels of interleukin‐6 and tumor necrosis factor‐α—which can contribute to tumorigenesis (34, 35). In addition, stress-related activation of the hypothalamic–pituitary–adrenal (HPA) axis and sympathetic nervous system (SNS) can modify the expression of cytokine–cytokine receptor interaction pathways in key brain regions like the amygdala, further linking neuroendocrine changes to inflammatory responses (36). These observations suggest that part of the genetic overlap observed between AD and HNC may be explained by shared molecular pathways that mediate neuroendocrine and immune responses under conditions of chronic psychological stress.

In the context of HNC, the inflammatory response is a critical driver of tumor progression by regulating angiogenesis, cell proliferation, and metastasis (37). Stress-induced hormonal alterations can modify the tumor microenvironment by enhancing the production of pro-inflammatory cytokines and growth factors, thereby creating conditions that favor tumor growth and dissemination (38). Together, these findings underscore the complex interplay between psychological stress, inflammation, and cancer biology, and highlight the need for further investigation into the specific molecular mechanisms linking AD to HNC.

Chronic inflammation in HNC can intensify anxiety symptoms by disrupting neural circuits that regulate stress responses, potentially creating a feedback loop that worsens clinical outcomes. For example, inflammatory cytokines may alter neurotransmitter systems involved in mood regulation, thereby increasing anxiety levels in HNC patients. Targeting these inflammatory pathways could offer a novel therapeutic strategy to alleviate anxiety and improve quality of life in this population (9). To note, this interplay may be influenced by behavioral and environmental factors, such as smoking or stress-related immune suppression, which can modulate inflammatory pathways and exacerbate both anxiety and cancer progression (39).

Emerging evidence suggests that transcriptional dysregulation may underlie both oncogenic processes in HNC and aspects of neurodevelopment relevant to ADHD. For instance, the MYC oncogene—frequently amplified in HNC (40)—is well known for its role in driving cell proliferation and tumor progression. Although the direct contribution of MYC to ADHD pathophysiology remains to be fully elucidated, recent studies indicate that aberrant MYC-related transcriptional programs can impact neurodevelopment and may be linked to behavioral phenotypes observed in ADHD (41). Additionally, research has identified various genes associated with ADHD, such as Cadherin-13 (CDH13), which impacts synaptic function and is implicated in neurodevelopmental processes relevant to ADHD (42). Similarly, studies have shown that the MYC gene is frequently amplified in head and neck squamous cell carcinoma (HNSCC), contributing to tumor progression (43). These findings suggest that shared genetic factors may underlie the co-occurrence of these conditions.

In addition to transcriptional regulators, the enzyme CYP2D6 plays a critical role in the metabolism of many psychostimulant and nonstimulant medications used in ADHD treatment. Polymorphisms in CYP2D6 can lead to considerable variability in drug metabolism, as evidenced by its established impact on the pharmacokinetics of atomoxetine (44). While CYP2D6 genetic variations have been linked to altered susceptibility to HNC (45), it is important to note that there is no direct evidence suggesting a shared mechanism that would cause ADHD itself. The dual relevance of CYP2D6 in ADHD medication efficacy and cancer risk reflects complex interactions between genetic factors and environmental exposures but does not imply a single, common mechanism underlying the development of ADHD.

## Advantage

The study employs an AI-based computational approach to identify disease-gene associations, which allows for the analysis of a vast number of genes (19,924) and the identification of significant overlaps among AD, ADHD, and HNC. The use of Fisher’s exact test and odds ratios provides robust statistical validation of these overlaps, highlighting shared genetic factors across these conditions. The integration of PPI network analysis further elucidates the connectivity and functional roles of key genes, offering insights into potential shared pathways and molecular mechanisms underlying these diseases.

## Limitation

This study examines genetic overlaps between psychiatric disorders and HNC, highlighting the distinct diagnostic criteria and biological mechanisms of these conditions. While data mining offers valuable insights, it is important to acknowledge the potential pitfalls of analyzing large, non-specific datasets. Without rigorous data curation and validation, the risk of misinterpretation increases, which could lead to false associations. As noted in a review on clinical data mining, “the exotic predictions of data mining are difficult to apply directly in local medical institutions” (46).

Additionally, the study’s reliance on existing literature for gene-disease associations introduces potential bias, as it depends on the availability and quality of prior research. The focus on statistical significance may overlook biologically relevant genes with smaller effect sizes. Furthermore, the absence of experimental validation of the identified gene associations limits the direct applicability of these findings to clinical settings.

While shared genetic pathways have been identified between psychiatric disorders and HNC, the biological significance of these overlaps remains to be fully elucidated. The complexity of gene expression and regulation in both conditions suggests that these shared pathways may operate differently across tissues and contexts. Gene-environment interactions and behavioral factors could further influence these findings, highlighting the need for a comprehensive understanding of how environmental factors may shape the genetic underpinnings of these conditions.

Therefore, further research is imperative to understand the functional implications of these genetic overlaps and their potential impact on disease pathogenesis and treatment strategies. In conclusion, while the identification of common genetic factors between psychiatric disorders and HNC is a promising avenue for research, it is crucial to approach these findings with a critical perspective. Recognizing the diagnostic differences and the limitations inherent in data mining methodologies will ensure that future studies yield meaningful and clinically relevant insights.

## Conclusion

This study uncovers a complex and interconnected genetic network among AD, ADHD, and HNC, highlighting shared pathways, unique mechanisms, and critical genes. These findings provide valuable insights into the genetic underpinnings of these conditions and open potential avenues for therapeutic exploration. Furthermore, understanding these genetic connections could guide future research into targeted interventions and inform clinical practice by identifying new biomarkers and therapeutic targets for these disorders.

## Data Availability

The original contributions presented in the study are included in the article/**Supplementary Material**. Further inquiries can be directed to the corresponding authors.
