# Supplementary material for: Gene-level connections between anxiety disorders, ADHD, and head and neck cancer: insights from a computational biology approach
Source: Front Psychiatry. 2025 Mar 20;16:1552815. doi: 10.3389/fpsyt.2025.1552815 (PMC11967369; doi:10.3389/fpsyt.2025.1552815)
Supplement: Supplementary file 1 [file DataSheet1.zip › Python pakage/Help files.docx]

**Packages needed for our package**

**==========================================================**

**Packages needed:**

pip install pandas

pip install sklearn

pip install scikit-learn

pip install IPython

pip install pattern

pip install Bio

pip install openai

pip install networkx

pip instal xlrd

pip install openpyxl

**Note:**

1. *To install pattern:*

sudo yum install mysql-devel

sudo yum install python3-devel

pip install mysqlclient

**Install Python 3.10:**

https://medium.datadriveninvestor.com/installing-python-3-10-and-flask-on-godaddy-updated-ebe764ab78a7

./configure --prefix=/usr/local/python3.10 --enable-shared LDFLAGS="-Wl,-rpath /usr/local/lib"

#chage permission of a folder and sub-folder and files

Cd software

#create virtual envirenment

python3 -m pip install virtualenv

virtualenv FolderName

source PythonTest/bin/activate

- **To install db4-devel**

wget <https://public-yum.oracle.com/public-yum-ol6.repo> -O /etc/yum.repos.d/public-yum-ol6.repo

yum install --nogpgcheck -y db4-devel

- **To create a file and edit it:**
  touch myfile.txt

nano myfile.txt

- **For error: /root/Python-3.10.5/Modules/_ctypes/_ctypes.c:107:10: fatal error: ffi.h: No such file or directory #include <ffi.h>**

sudo yum install libffi-devel

- **make sue sQlite is supported!**

./configure --prefix=$HOME/.local/ --enable-sqlite-fts5

./configure --prefix=$HOME/.local/ --enable-shared LDFLAGS="-Wl,-rpath $HOME/.local/lib" --enable-sqlite-fts5mak

**install python:**

**https://www.godaddy.com/resources/skills/how-to-install-and-configure-python-on-a-hosted-server**

**https://sysally.com/general/install-python-3-x-whm-cpanel-server**

**https://medium.datadriveninvestor.com/installing-python-3-10-and-flask-on-godaddy-updated-ebe764ab78a7**

=============================

chmod +x configure

./configure --prefix=$HOME/.local --with-openssl=$HOME/.local/

--enable-shared LDFLAGS="-L$HOME/.local/lib64 -Wl,

--rpath=$HOME/.local/lib" CPPFLAGS="-I$HOME/.local/lib/libffi-3.2.1/include" CFLAGS="-I$HOME/.local/lib/libffi-3.2.1/include" PKG_CONFIG_PATH="$HOME/.local/lib/pkgconfig" LD_LIBRARY_PATH=${LD_LIBRARY_PATH}:$HOME/.local/lib
