# Supplementary material for: Gene-level connections between anxiety disorders, ADHD, and head and neck cancer: insights from a computational biology approach
Source: Front Psychiatry. 2025 Mar 20;16:1552815. doi: 10.3389/fpsyt.2025.1552815 (PMC11967369; doi:10.3389/fpsyt.2025.1552815)
Supplement: Supplementary file 1 [file DataSheet1.zip › Python pakage/readme.docx]

**Introduction**

This code provides functions for finding all possible paths in a directed graph, removing subpaths from a list of paths, and summarizing information using OpenAI API.

**Dependencies**

The code requires the following dependencies:

- pandas
- networkx
- openai

Please make sure these libraries are installed before running the code.

**Example Usage**

Here's an example of how to use the functions in this code:

import tnetworkx

import UsefulTools

import pandas as pd

UsefulTools.clear_console()

### Load data from a Excel file

refTablefilePath=r'Example_Ref.xlsx'

savePath=r'r_all_paths.csv'

df_ref = pd.read_excel(refTablefilePath)

ref = tnetworkx.preprocess(df_ref)

all_paths = tnetworkx.process(ref)

### Save the result to a csv file

summaryLength=50

tnetworkx.summary_from_openai(all_paths, savePath, summaryLength)

Make sure to replace **"your_api_key"** with your actual OpenAI API key and provide appropriate input data for the other functions.

**Usage**

**find_all_paths(node1_list, node2_list)**

This function returns all possible paths in a directed graph created from two lists of nodes. The input parameters are **node1_list** and **node2_list**, which represent two lists of nodes. Each point in **node1_list** is connected to the corresponding point in **node2_list**. The output is a list of all possible paths in the graph.

Input: two lists of strings node1_list and node2_list

Output: A list where each element is a list of all nodes on a path.

First build a directed graph by adding nodes and each line segment, then collect all possible paths in the graph into a list (all_paths), and return this list.

**remove_subpaths(all_paths)**

This function removes subpaths from a list of paths. The input parameter is **all_paths**, which is a list of paths. It modifies the **all_paths** list in-place by removing any subpaths.

Input: A list (all_paths) where each element is a list representing a path.

output: none

Concatenate each path in all_paths into a string with '->', so that each element in the new list temp is a path string. If a string in temp is a substring of another string in temp, remove the corresponding path from all_paths.

**openai_response(m)**

This function generates a response using the OpenAI API. The input parameter **m** is a message string. The function sets the OpenAI API key and uses the OpenAI Completion API to generate a response based on the provided prompt. The output is the text response from OpenAI or 'Error' if an error occurs.

Input: a string (m).

Output: Returns the response from OpenAI, or an empty string if an error occurred.

Call OpenAI's API, return OpenAI's response or an empty string. It may be necessary to submit some input parameters, such as: api_key, max_tokens, etc.

**preprocess(df, f='')**

This function preprocesses a dataframe by extracting entity names, selecting references, creating a polarity column, appending PMID/DOI to sentences, and saving the data to a file. The input parameter **df** is a dataframe with columns: **Relation Name**, **Sentence**, **PMID**, and **DOI**. The optional parameter **f** is a csv file to save the resulting data. The output is a dataframe with additional columns.

Input: a dataframe with 'Relation Name', 'Sentence', 'PMID', and 'DOI'.

Output: a dataframe with ‘Entity1 Name’, ‘Entity2 Name’, ‘polarity’, and ‘SPD’.

1. From 'Relation Name', 'Entity1 Name' and 'Entity2 Name' can be extracted.
2. If there are multiple records for the same 'Relation Name', select the latest 5 records.
3. 'polarity' can be extracted from ‘Relation Name’.
4. Combine 'Sentence' and 'PMID/DOI' for each record.
5. Processed data can be saved to a file (optional).

**process(df)**

This function processes a dataframe by getting all possible paths, creating a dataframe to store path information, and retrieving polarity and sentences for each path. The input parameter **df** is a dataframe with columns **Entity1 Name** and **Entity2 Name**. The output is a dataframe with start, target, paths, polarity, and sentences.

Input: DataFrame with "Entity 1 Name" and "Entity 2 Name"

Output: DataFrame with 'start', 'target', 'polarity', 'sentence_length' and 'sentence'

1. Delete duplicate pairs of "Entity1 Name" and "Entity2 Name", construct all possible paths, keep paths with length less than 4, and delete all subpaths.

2. Extract 'start' and 'target', and keep the entire path.

3. Integrate 'polarity' and 'SPD' of each segment to form 'polarity', 'sentences', and 'length of sentences' of a path.

**summary_from_openai(all_paths, f='', summaryLength=60, debug=True)**

This function retrieves responses from OpenAI for each path and generates summaries based on the provided sentences. The input parameter **all_paths** is a dataframe containing start, target, paths, polarity, and sentences. The optional parameter **f** is a csv file to save the results. The **summaryLength** parameter specifies the maximum length of the summary text in words. The **debug** parameter controls whether progress and debug information is displayed during execution.

Input: all_paths (DataFrame with "start", "target" and "sentences"), f (file to save summary), summaryLength (length of summary), debug (whether to display debug information).

Output: None

From each input record, extract 'start', 'target', and 'sentences' in advance, add prompt information, then submit to ChatGPT, and save the obtained summary to 'response from chatgpt'. It can be saved to a file (optional).
