# Supplementary figures and images for: Gene-level connections between anxiety disorders, ADHD, and head and neck cancer: insights from a computational biology approach
Source: Front Psychiatry. 2025 Mar 20;16:1552815. doi: 10.3389/fpsyt.2025.1552815 (PMC11967369; doi:10.3389/fpsyt.2025.1552815)

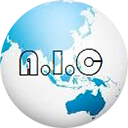

Supplement: Supplementary file 1 [file DataSheet1.zip › Python pakage/logo_ico.png]
